# Pre-hospital advanced airway management by anaesthesiologists: Is there still room for improvement?

**DOI:** 10.1186/1757-7241-16-2

**Published:** 2008-07-21

**Authors:** Stephen JM Sollid, Jon Kenneth Heltne, Eldar Søreide, Hans Morten Lossius

**Affiliations:** 1 Department of Anaesthesia and Intensive care, Division of Acute Care Medicine, Stavanger University Hospital, Stavanger, Norway; 2 Department of Anaesthesia and Intensive Care, Haukeland University Hospital, Bergen, Norway; 3 Department of Research and Development, Norwegian Air Ambulance Foundation, Drøbak, Norway

## Abstract

**Background:**

Endotracheal intubation is an important part of pre-hospital advanced life support that requires training and experience, and should only be performed by specially trained personnel. In Norway, anaesthesiologists serve as Helicopter Emergency Medical Service HEMS physicians. However, little is known about how they themselves evaluate the quality and safety of pre-hospital advanced airway management.

**Method:**

Using a semi-structured questionnaire, we interviewed anaesthesiologists working in the three HEMS programs covering Western Norway. We compared answers from specialists and non-specialists as well as full- and part-time HEMS physicians.

**Results:**

Of the 17 available respondents, most (88%) felt that their continuous exposure to intubations was not sufficient. Additional training was mainly acquired through other clinical practice and mannequin- or cadaver-based skills training. Of the respondents, 77% and 35% reported having experienced difficult and failed intubations, respectively. Further, 59% reported knowledge of airway management-related deaths in their HEMS program. Significantly more full- than part-time HEMS physicians had experienced these problems. All respondents had airway back-up equipment in their service, but 29% were not familiar with all the equipment.

**Conclusion:**

The majority of anaesthesiologists working as HEMS physicians view pre-hospital advanced airway management as a high-risk procedure. Relevant airway management competencies for HEMS physicians in Norway seem to be insufficiently trained and maintained. A better-defined level of competence with better training methods and systems seems warranted.

## Background

Endotracheal intubation (ETI) plays an important role in pre-hospital advanced life support (ALS) [1-3]. Despite this fact, there is an increased concern that both quality of care and patient safety suffer from intubation attempts by pre-hospital clinicians with limited training and experience [4,5]. The notion that advanced airway management in the pre-hospital setting should only be handled by specially trained personnel has led to the recently developed guidelines for pre-hospital airway management by the Scandinavian Society for Anaesthesiology and Intensive care medicine (SSAI)[6]. These guidelines stress the importance of extensive airway management experience and the ability to use anaesthetic drugs to facilitate ETI, thus suggesting that the skill should be restricted to only anaesthesiologists [6]. As in other European countries, anaesthesiologists play an active role as Helicopter Emergency Medical System (HEMS) physicians in Norway [7]. Studies from other European countries have shown that intubation problems and complications are common also in anaesthesiologist-manned systems [8-10]. To the best of our knowledge, similar data are not available for Norway or other Scandinavian countries. We therefore wanted to survey anaesthesiologists working as HEMS physicians to see how they evaluate the quality and safety of their own pre-hospital airway management. Such a survey could create a basis for further risk management and quality improvement initiatives.

## Method

**Figure 1:**
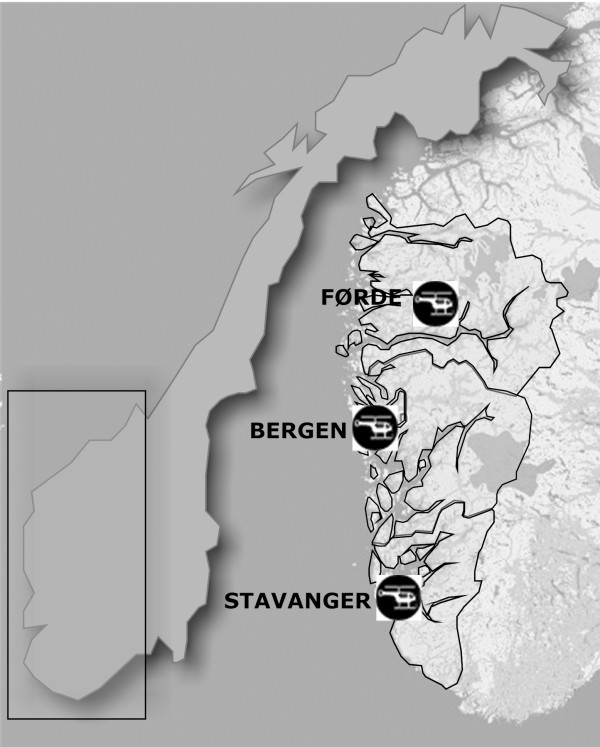
The three Western Norway counties of Sogn og Fjordane, Hordaland and Rogaland with their respective HEMS programs based in Førde, Bergen and Stavanger.

We interviewed anaesthesiologists working in the three Norwegian Air Ambulance HEMS programs covering Western Norway, a 43 000 km^2 ^region with a total of approximately 970 000 inhabitants and an overall population density of about 22 individuals per km^2 ^(Figure 1). In cases where weather or technical problems prohibit the use of helicopters, or where the scene is close to the HEMS base, the HEMS physician goes to the scene using a rapid response car (RRC) (Table 1) [7]. These three programs were chosen because we think they illustrate both the diversity of, and the similarities between, the Norwegian Air Ambulance services. They are based on the same "three-crew" concept and have the same operator, but they have primary response areas that are diverse in mission and population profile.

**Table 1:** Number of missions carried out by the three HEMS programs during 2006 with helicopter and Rapid Response Car (RRC).

HEMS Base	Helicopter Missions	Rapid Response Car (RRC)	Total mission
Bergen	902 (1139)	501 (690)	1403 (1829)
Førde	725 (918)	86 (87)	811 (1309)
Stavanger	787 (847)	450 (459)	1237 (1005)

The corresponding number of mission requests is in parentheses.

We used a semi-structured questionnaire in Norwegian (for a translated version, see Additional file 1) with mainly fixed-response questions like yes/no and multiple-choice. In selected questions we allowed for comments depending on the response given. The questionnaire was developed by two of the authors (SS and JKH) for the purpose of this study. Relevant activity data from the three programs were collected from the joint activity database "AirDoc" to identify the actual volume of advanced airway management in the programs.

The results were recorded in a FileMaker Pro database (FileMaker Inc., Santa Clara, CA, USA) and analysed using SPSS (SPSS Inc., Chicago, IL, USA).

Since the activity data used in this study are officially available in annual reports and all questions were answered voluntarily, it was not deemed necessary to seek approval from the Regional Ethics Committee for this study. The results were compared using Fischer's Exact test. A p-value < 0.05 was considered statistically significant.

## Results

In 2006, the three HEMS programs overall completed 3451 missions (Table 1). Seventeen of the 20 anaesthesiologists in the three programs were interviewed; the remaining three reside outside of Norway and were not available for the study.

**Figure 2:**
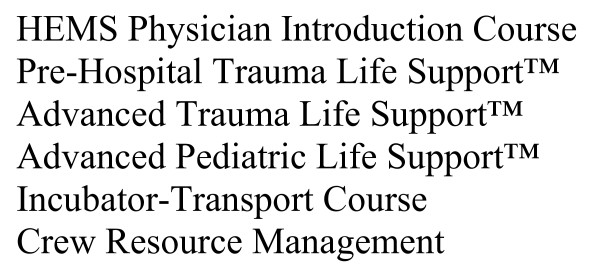
Courses that are recommended and relevant for HEMS physicians in Norway.

The majority (71%) of the anaesthesiologists working as HEMS physicians were certified specialists (Table 2). Only one (6%) had attended all the recommended courses for HEMS Physicians within the last four years (Figure 2), while five (29%) had attended all the recommended Life Support (LS) courses (Table 2). Respondents' experience in years within anaesthesiology and as HEMS physicians, and number of respondents who attended Life Support (LS) courses during the last four years, differentiated between specialists and non-specialists in anaesthesiology.

**Table 2:** Respondents' experience in years within anaesthesiology and as HEMS physicians, and number of respondents who attended Life Support (LS) courses during the last four years, differentiated between specialists and non-specialists in anaesthesiology.

	Anaesthesiology (years)			HEMS physician (years)			Attended LS Courses last 4 yrs		
	
	Median	Min	Max	Median	Min	Max	PHTLS	ATLS	APLS
Specialist (n = 12)	14,5	6,0	29,0	10,0	1,5	25,0	8	4	5
Non-specialist (n = 5)	4,0	2,5	5,0	1,0	1,0	5,0	3	5	3
All (n = 17)	11,0	2,5	29,0	8,0	1,0	25,0	11	9	8

All but two respondents felt they needed a certain volume of ETIs to maintain their airway management skills. The desired number of ETIs per month ranged from five to fifteen (median 5), but only one HEMS physician (6%) said he achieved this goal. The number of actual ETIs encountered per month ranged from one to ten (median 2).

Thirteen (77%) of the 17 HEMS physicians reported having experienced a difficult airway situation in the pre-hospital setting, and six respondents (35%) had experienced a failed pre-hospital ETI. More full- than part-time HEMS physicians reported these airway problems (p = 0.006 and p = 0.043, respectively) (Table 3). Most frequently, ETI difficulties were reported in trauma patients. Ten (59%) of the physicians in the survey had knowledge of deaths following pre-hospital ETI complications in their own HEMS (Table 3).

**Table 3:** Respondents reported experience with difficult airway situations and difficult airway back-up equipment.

	Specialist (n = 12)	Non specialist (n = 5)	P	Fulltime (n = 11)	Part-time (n = 6)	P
Has experienced difficult airway	10	3	0.538	**11**	**2**	**0.006**
Has experienced non-intubation situation	6	0	0.102	**6**	**0**	**0.043**
Has knowledge of airway related death	7	3	1.000	6	4	1.000
Experience with use of ILMA	6	4	0.338	5	6	0.304
Experience with use of LMA	7	4	0.600	6	5	0.333
Experience with use of LMA Proseal	4	3	0.593	3	4	0.162
Experience with use of LT	5	1	0.600	**5**	**6**	**0.043**
Experience with use of McCoy	5	2	1.000	5	2	1.000
Experience with use of Bougie	7	4	0.600	6	5	0.333
Experience with use of needle cricothyrotomy	4	0	0.261	4	0	0.237
Experience with use of cricothyrotomy	3	0	0.515	3	0	0.515
Experience with use of emergency tracheotomy	2	0	1.000	2	0	0.515

Significant differences are in bold (p-value < 0.05 considered significant).

All HEMS programs had back-up equipment for difficult airway management. The most preferred backup devices were the Intubating Laryngeal Mask Airway (ILMA), the Laryngeal Tube (LT) and the Gum Elastic Bougie (Table 3). Significantly more part-time HEMS physicians than full-time HEMS physicians had experience with the LT (p = 0.043) (Table 3). Two respondents had no training in the use of the Gum Elastic Bougie, although it was part of their airway-backup kit. The same was true for three respondents regarding the use of trans-tracheal techniques (needle and emergency cricothyrotomy). The other 14 (82%) physicians had been trained in the use of trans-tracheal techniques, but only five, all of whom were specialists and full-time HEMS physicians, had any experience with the use of one or more of them.

When asked how they maintained their own advanced airway skills, all but one had some strategy for this. Six respondents relied solely on the experience gained in their work as anaesthesiologists or HEMS physicians, while the other 10 (59%) combined this with training on manikins (n = 7), training on cadavers (n = 5) or attending airway management courses (n = 3). Only one reported experience with high fidelity patient simulators for this purpose.

## Discussion

There seems to be a need for continuous ETI skills maintenance and difficult airway management training among anaesthesiologists working as HEMS physicians in Norway. While airway management problems do occur, not all of the physicians seem prepared or properly trained to handle them. Our survey indicates that, although the HEMS physicians felt they needed training to maintain their advanced airway management proficiency, this was left to the individual physician to organize.

One limitation of our survey is that it covered only three HEMS programs in one region of Norway. We still believe that the findings are representative for other HEMS programs in Norway since these other programs are organized in a similar fashion. However, beyond Norway, it is more difficult to generalize based on our findings, because physician-manned HEMS in other countries are organized differently and may have different systems for training and maintaining advanced airway management skills. Despite this, we believe that the problems addressed here are applicable to other HEMS systems manned with anaesthesiologists [10,11]. Further, surveys are prone to bias, especially when attitudes are surveyed [12]. We do, however, believe that our sample population is representative because it comes from three programs and the response rate was 85%. Also, we have tried to minimize instrument bias by using mostly fixed response questions [12].

Regarding the statistical analysis, the significance tests should be interpreted with caution because the sample set is limited. Still, we believe the differences that were uncovered are valid as indicators of how anaesthesiologists themselves view the risks associated with pre-hospital airway management.

There are currently 11 HEMS programs [13] in Norway. HEMS physicians are recruited and employed by the local Health Trusts and must have at least two years of practice within the speciality of anaesthesiology. In addition, all residents have completed a 1.5-year internship including internal medicine, general surgery and primary health service before entering a residency program. This minimum level of competence is defined in two Norwegian governmental reports [13,14] and is the only official statement on what competence is required for HEMS physicians [7]. A dedicated HEMS introduction course and LS courses are recommended (Figure 2), but it is left to the employer to include this in the job requirements or to the individuals to participate based on their own initiative. Thus, there are potentially as many different ways to ensure the proper competence of HEMS physicians as there are employers or programs.

It is well documented that there is a certain learning curve involved with critical skills in the speciality of anaesthesiology, including ETI [15,16]. When it comes to retaining these skills, it is proposed that a certain number of procedures must be performed regularly [16], but little is known about the volume and regularity of the repetition of these skills. In our survey, almost all anaesthesiologists in HEMS felt that they needed a certain volume of cases to maintain their intubation skills. In a pre-hospital environment it is, however, hard to meet the expectation of 10 or more intubations per month, or even 10 per year as cited by others [6]. During 2006, 264 patients were intubated outside the hospital while being cared for by the three HEMS programs (data from HEMS Activity Database "AirDoc"). This means an average of 22 intubations per month for all three HEMS programs, shared among almost 20 physicians. If the desired number of intubations is to be met, other sources of airway management training must be found in addition to pre-hospital clinical experience. HEMS physicians could gain more experience if they combined their work with hospital work in the intensive care unit, the emergency department or the operating room. This kind of rotation does not, at present, seem to be standardized. For HEMS physicians this would have other obvious benefits; for example the chance to train on other emergency medicine-related skills. Still, clinical practice alone is no guarantee that the desired level of skills proficiency in advanced and difficult airway management would be acquired and maintained [11,17,18].

The frequency of airway management complications is probably lower in physician manned pre-hospital services than in non-physician manned pre-hospital services [3,8], but at the same time, studies have shown that anaesthesiologists are probably not as well prepared for difficult airway situations as expected [11,17-19]. Our study did show that the majority had experience with severe complications and knew of deaths in their own system. Some respondents also reported inadequate training on, knowledge of and experience with their own airway management back-up equipment. With a low volume of actual ETIs and inadequate training opportunities for advanced airway management, including Crisis Resource Management (CRM) [20], we think that the HEMS physicians are not optimally prepared for advanced pre-hospital airway management.

It has been reported that more than 80% of HEMS physicians in Norway are specialists in anaesthesiology [14]. According to our research, this is still valid. We did not find any significant differences in experienced airway problems when comparing specialists to non-specialists (Table 3). Still, six of twelve specialists had experienced non-intubation situations, but none of the five non-specialists. We also compared full- and part-time HEMS physicians, and found significant differences in the amount of experience dealing with airway problems (Table 3). This probably mirrors the difference in their caseload, but it could also be used as an argument for full-time employment of specialists, as this might ensure more exposure to challenges relevant to the job.

In recent years, training in full-scale medical simulators has emerged as a new way of training health-professionals. Successful airway management curricula have been created [21], also for HEMS services [22]. Some HEMS programs have established training and certification systems using low fidelity simulation [23]. Others have introduced mandatory simulation practice for all HEMS medical crew (preliminary report by Gerson et al., International Meeting on Simulation in Healthcare, San Diego, USA, 2008). However, the patient simulators still are not realistic enough to fully replace the real patient as a training object when it comes to advanced airway management [24]. However, if the focus is on training strategies to handle complications in airway management, or CRM [20], the use of full-scale simulation and patient simulators seems effective [22,25]. If the goal is to ensure uniform quality of care from all HEMS physicians, simulation could probably also play a role in individualizing the learning and training experience for the individual physician [26].

Also, from a patient safety perspective, we think it is important to better define what competence HEMS physicians should have and establish better routines for training and retaining critical skills like advanced airway management. However, further studies are needed to better quantify the hazards and risks that patients are exposed to in the current system and to tailor future training and continuing educational programs for HEMS physicians.

## Conclusion

Relevant airway management competencies for HEMS physicians in Norway seem to be insufficiently trained and maintained. A better-defined competency level for HEMS physicians seems warranted. Further studies are needed to determine how new training methods can improve the airway management competence of HEMS physicians and to what extent this will improve outcome.

## Competing interests

The authors declare that they have no competing interests.

## Authors' contributions

SJMS conceived the study and designed the questionnaires, carried out halve of the interviews, managed the data and carried out the statistics and drafted the manuscript. JKH participated in the design of the study and the questionnaires, carried out halve the interviews and helped to draft the manuscript. ES helped conceive the study and helped to draft the manuscript. HML helped conceive the study and helped to draft the manuscript.

## Supplementary Material

Additional file 1 Translated questionnaire. English translation of the Norwegian questionnaire used during interviews. Click here for file

## References

[B1] Nolan JP, Deakin CD, Soar J, Bottiger BW, Smith G (2005). European Resuscitation Council guidelines for resuscitation 2005. Section 4. Adult advanced life support. Resuscitation.

[B2] Smith CE, Walls RM, Lockey D, Kuhnigk H, Søreide E, Grande CM (2001). Advanced airway management and use of anesthetic drugs. Prehospital trauma care.

[B3] Klemen P, Grmec S (2006). Effect of pre-hospital advanced life support with rapid sequence intubation on outcome of severe traumatic brain injury. Acta Anaesthesiol Scand.

[B4] Davis DP, Fakhry SM, Wang HE, Bulger EM, Domeier RM, Trask AL, Bochicchio GV, Hauda WE, Robinson L (2007). Paramedic rapid sequence intubation for severe traumatic brain injury: perspectives from an expert panel. Prehosp Emerg Care.

[B5] Timmermann A, Russo SG, Eich C, Roessler M, Braun U, Rosenblatt WH, Quintel M (2007). The out-of-hospital esophageal and endobronchial intubations performed by emergency physicians. Anesth Analg.

[B6] Berlac P, Hyldmo PK, Kongstad P, Kurola J, Rostrup Nakstad A, Sandberg M (2008). Prehospital airway management – guidlines from a task force from the Scandinavian society for anaesthesiology and Intensive care Medicine. Acta Anaesthesiol Scand.

[B7] Langhelle A, Lossius HM, Silfvast T, Bjornsson HM, Lippert FK, Ersson A, Soreide E (2004). International EMS Systems: the Nordic countries. Resuscitation.

[B8] Adnet F, Jouriles NJ, Le Toumelin P, Hennequin B, Taillandier C, Rayeh F, Couvreur J, Nougiere B, Nadiras P, Ladka A, Fleury M (1998). Survey of out-of-hospital emergency intubations in the French prehospital medical system: a multicenter study. Ann Emerg Med.

[B9] Thierbach A, Piepho T, Wolcke B, Kuster S, Dick W (2004). [Prehospital emergency airway management procedures. Success rates and complications]. Anaesthesist.

[B10] Timmermann A, Eich C, Russo SG, Natge U, Brauer A, Rosenblatt WH, Braun U (2006). Prehospital airway management: a prospective evaluation of anaesthesia trained emergency physicians. Resuscitation.

[B11] Rosenstock C, Ostergaard D, Kristensen MS, Lippert A, Ruhnau B, Rasmussen LS (2004). Residents lack knowledge and practical skills in handling the difficult airway. Acta Anaesthesiol Scand.

[B12] Rubenfeld GD (2004). Surveys: an introduction. Respir Care.

[B13] St. meld. nr 43 (1999–2000). Om akuttmedisinsk beredskap. [Oslo]: Sosial-og Helsedepartementet.

[B14] (1998). NOU (1998:8). Luftambulansetjenesten i Norge. NOU 1998:8. Oslo: Statens forvaltningstjeneste.

[B15] de Oliveira Filho GR (2002). The construction of learning curves for basic skills in anesthetic procedures: an application for the cumulative sum method. Anesth Analg.

[B16] Konrad C, Schupfer G, Wietlisbach M, Gerber H (1998). Learning manual skills in anesthesiology: Is there a recommended number of cases for anesthetic procedures?. Anesth Analg.

[B17] Rosenstock C, Hansen EG, Kristensen MS, Rasmussen LS, Skak C, Ostergaard D (2006). Qualitative analysis of unanticipated difficult airway management. Acta Anaesthesiol Scand.

[B18] Kristensen MS, Moller J (2001). Airway management behaviour, experience and knowledge among Danish anaesthesiologists – room for improvement. Acta Anaesthesiol Scand.

[B19] Mort TC (2004). Emergency tracheal intubation: complications associated with repeated laryngoscopic attempts. Anesth Analg.

[B20] Rall M, Dieckmann P (2005). Safety culture and crisis resource management in airway management: general principles to enhance patient safety in critical airway situations. Best Pract Res Clin Anaesthesiol.

[B21] Rosenthal ME, Adachi M, Ribaudo V, Mueck JT, Schneider RF, Mayo PH (2006). Achieving housestaff competence in emergency airway management using scenario based simulation training: comparison of attending vs housestaff trainers. Chest.

[B22] Davis DP, Buono C, Ford J, Paulson L, Koenig W, Carrison D (2007). The effectiveness of a novel, algorithm-based difficult airway curriculum for air medical crews using human patient simulators. Prehosp Emerg Care.

[B23] Bredmose P (2007). London's Air Ambulance – HEMS. Scand J Trauma Resusc Emerg Med.

[B24] Hesselfeldt R, Kristensen MS, Rasmussen LS (2005). Evaluation of the airway of the SimMan full-scale patient simulator. Acta Anaesthesiol Scand.

[B25] Nargozian CD (2004). Simulation and airway-management training. Curr Opin Anaesthesiol.

[B26] Issenberg SB, McGaghie WC, Petrusa ER, Lee Gordon D, Scalese RJ (2005). Features and uses of high-fidelity medical simulations that lead to effective learning: a BEME systematic review. Med Teach.

