# Supplementary material for: Pre-hospital advanced airway management by anaesthesiologists: Is there still room for improvement?
Source: Scand J Trauma Resusc Emerg Med. 2008 Jul 21;16:2. doi: 10.1186/1757-7241-16-2 (PMC2556637; doi:10.1186/1757-7241-16-2)
Supplement: Additional file 1 — Translated questionnaire. English translation of the Norwegian questionnaire used during interviews. [file 1757-7241-16-2-S1.doc]

Additional file 1

| Certified Anaesthesiologist? | ☐ Yes ☐ No | |
| --- | --- | --- |
| Experience in Anaesthesiology? | ____________ years | |
| Experience pre-hospital? | ____________ years | |
| Do you work pre-hospital full-time? If not, estimated % of work pre-hospital. | ☐ 100% ☐ _____% | |
| Have you taken any of these courses within the last four years? | ☐ PHTLS ☐ ATLS  ☐ APLS ☐ Incubator transport course  ☐ HEMS physician introduction course  ☐ CRM ☐ SSAI Airway course | |
| Have you taken any other relevant advanced airway courses in the same period? | ______________________________  ______________________________ | |
| How many endotracheal intubations do you yourself perform pre-hospital each month? (Estimated number) | ____________ | |
| Do you think that you need a certain number of endotracheal intubations (pre- and intrahospital) to maintain the skill? If yes, how many? If no, why not? | ☐ Yes ☐ No  ______________________________  ______________________________  ______________________________ | |
| Have you experienced a difficult airway situation pre-hospital?  (Definitions: >2 attempts/ >2 minutes/ no direct view of larynx/ oesophageal intubation) | ☐ Yes  ☐ No | If yes, patient category  ☐ Traumapatient ☐ Cardiac arrest  ☐ Respiratory failure ☐ Pediatric  ☐ Other __________ |
| Have you experienced a failed intubation attempt pre-hospital? | ☐ Yes  ☐ No | If yes, patient category  ☐ Traumapatient ☐ Cardiac arrest  ☐ Respiratory failure ☐ Pediatric  ☐ Other __________ |
| Do you have knowledge of deaths related to airway management problems in your program? | ☐ Yes ☐ No | |
| Does your program have equipment for the management of airway problems?  If yes, what equipment? | ☐ Yes ☐ No ☐ Don’t know  Supraglottic  ☐ LMA ☐ LMA Proseal ☐ ILMA  ☐ Larynxtube ☐ Combitube  ☐ Other ____________________  ETT assist devices  ☐ Mc Coy laryngoscope ☐ Bougie  ☐ Other ____________________  Trans-tracheal  ☐ Needlecricothyrotomy w/Jet ventilation  ☐ Cricothyrotomi ☐ Emergency Trach  ☐ Other ____________________ | |
| Have you received training or trained yourself in the use of any of these tools for the management of a difficult airway? | Supraglottic  ☐ LMA ☐ LMA Proseal ☐ ILMA  ☐ Larynxtube ☐ Combitube  ☐ Other ____________________  ETT assist devices  ☐ Mc Coy laryngoscope ☐ Bougie  ☐ Other ____________________  Trans-tracheal  ☐ Needlecricothyrotomy w/Jet ventilation  ☐ Cricothyrotomi ☐ Emergency Trach  ☐ Other ____________________ | |
| Do you have experience in the use of any of these tools for the management of a difficult airway? | Supraglottic  ☐ LMA ☐ LMA Proseal ☐ ILMA  ☐ Larynxtube ☐ Combitube  ☐ Other ____________________  ETT assist devices  ☐ Mc Coy laryngoscope ☐ Bougie  ☐ Other ____________________  Trans-tracheal  ☐ Needlecricothyrotomy w/Jet ventilation  ☐ Cricothyrotomi ☐ Emergency Trach  ☐ Other ____________________ | |
| What is your preferred equipment in a “can ventilate – can’t intubate” situation? | Supraglottic  ☐ LMA ☐ LMA Proseal ☐ ILMA  ☐ Larynxtube ☐ Combitube  ☐ Other ____________________  ☐ BVM ☐ Pocketmask ☐ Guidel airway  ETT assist devices  ☐ Mc Coy laryngoscope ☐ Bougie  ☐ Other ____________________ | |
| What is your preferred equipment in a “can’t ventilate – can’t intubate” situation? | Supraglottic  ☐ LMA ☐ LMA Proseal ☐ ILMA  ☐ Larynxtube ☐ Combitube  ☐ Other ____________________  Transtracheal  ☐ Needlecricothyrotomy w/Jet ventilation  ☐ Cricothyrotomi ☐ Emergency Trach  ☐ Other ____________________ | |
| How do you maintain your competence and skills in managing difficult airways? | ☐ through regular clinical practice  ☐ training on manikins  ☐ training on corpses  ☐ through simulator training  ☐ through courses  ☐ other ____________________ | |
| What induction agents do you prefer for a RSI in trauma patients? | Sleep  ☐ Thiopenthone ☐ Diazepam☐ Midazolam ☐ Propofol  Analgesic  ☐ Morphine ☐ Fentanyl ☐ Alfentanil ☐ Pethidin  Neuromuscular blocking agent  ☐ Succinylcholine  ☐ Rocuronium ☐ Vecuronium ☐ Mivacurium  ☐ Cis-atracurium  Other  ☐ Ketamine ☐ __________________________ | |
| What induction agents do you prefer for a RSI in patients with isolated head-trauma? | Sleep  ☐ Thiopenthone ☐ Diazepam☐ Midazolam ☐ Propofol  Analgesic  ☐ Morphine ☐ Fentanyl ☐ Alfentanil ☐ Pethidin  Neuromuscular blocking agent  ☐ Succinylcholine  ☐ Rocuronium ☐ Vecuronium ☐ Mivacurium  ☐ Cis-atracurium  Other  ☐ Ketamine ☐ __________________________ | |
| Do you know if your program has written guidelines for airway management or the management of difficult airways pre-hospital? | ☐ Yes ☐ No ☐ Don’t know  ______________________________  ______________________________ | |
